# Gene–environment interactions in human health: case studies and strategies for developing new paradigms and research methodologies

**DOI:** 10.3389/fgene.2014.00271

**Published:** 2014-08-29

**Authors:** Fatimah L. C. Jackson

**Affiliations:** Department of Biology and W. Montague Cobb Research Laboratory, Howard UniversityWashington, DC, USA

**Keywords:** LP genetics, ASDs epigenetics, MetS, genomic–epigenomic-environment interactions, population substructure

## Abstract

The synergistic effects of genes and the environment on health are explored in three case studies: adult lactase persistence, autism spectrum disorders, and the metabolic syndrome, providing examples of the interactive complexities underlying these phenotypes. Since the phenotypes are the initial targets of evolutionary processes, understanding the specific environmental contexts of the genetic, epigenetic, and environmental changes associated with these phenotypes is essential in predicting their health implications. Robust databases must be developed on the local scale to deconstruct both the population substructure and the unique components of the environment that stimulate geographically specific changes in gene expression patterns. To produce these databases and make valid predictions, new, locally focused, and information-dense models are needed that incorporate data on evolutionary ecology, environmental complexity, local geographic patterns of gene expression, and population substructure.

## INTRODUCTION

The impact of gene–environment interactions in important human biological conditions affecting health are underappreciated due to a current imbalance in the knowledge needed to integrate and synthesize molecular, ecological, and sociocultural databases. In addition to the need for more precise data, there is also a great need for more specific interdisciplinary paradigms and research methodologies to identify and address these gene–environment interactions (Jackson, 2004). Environmental exposures of both high intensity and long duration are closely tied to health impacts. When these occur at the population level, such exposures can be of public health relevance. Even shorter exposures, however, can alter the signatures of epigenetic dysregulation in peripheral blood samples, within a few hours of environmental exposure (Baccarelli and Ghosh, 2012). Sustained behavioral and social processes and events can directly manipulate the epigenome and modulate the expression genome. Research on health risk assessment can benefit by developing ways to discern the differences in these effects.

A number of excellent examples exist of these kinds of interactions. We begin with the first case study of the genomic consequences among some proportion of humanity in response to generations of continued milk drinking beyond the weaning stage.

## CASE STUDY: ADULT LACTASE PERSISTENCE (LP): EVOLUTIONARY ECOLOGY, SUBSISTENCE PRACTICES, AND GENETIC DIVERSITY

Among almost all mammals, the ability to digest lactose declines sharply after infancy. High lactose digestion capacity in adult humans is common only in certain North Atlantic European, Mediterranean, Central Asian, and selected African populations, and overall, it is thought to be an evolutionary adaptation to millennia of drinking milk from domestic livestock (Holden and Mace, 1997). Prior to the domestication and use of milk producing species, there was no evolutionary advantage in the LP phenotype. Whereas afterward, there was and this has driven an entirely new dynamic in genetic evolution. When variability in the ability to digest and derive nutritional benefit from lactose was discovered among Western scientists, discussions of regular successful adult consumption of milk suggested that such practices were the human norm by using such terms as “adult lactose intolerance” and “adult lactase deficiency” to, in fact, describe what is the true human (and mammalian) behavioral and genetic norm. As the scope of the research was broadened to include non-European individuals and groups in the analyses as well as investigations among other mammals, it was realized that adult lactase persistence and adult lactose tolerance were the evolutionary exception. Something quite special had to have happened at a social, behavioral, and genetic levels in a small subset of humanity to allow adults to consume and derive nutritional benefit from the ingestion of animal milk without incurring the negative health consequences of lactose intolerance.

As the health-associated symptoms of the behavioral practice of adult milk drinking began to be examined by researchers with genetic training and evolutionary interests, we began to recognize that the practice of adult milk drinking had likely emerged during the early stages of animal domestication in many locales, and yet was geographically restricted to those groups with regular access to animal milk. The ability of human adults to successfully consume the milk of their domesticated livestock necessitated a genetic change in these humans, foremost of which was persistence of gene or genes for the production of the enzyme lactase into adulthood. LP is one of the clearest examples of niche construction in humans (Gerbault et al., 2011) where human modification of the environment significantly changes the direction of genetic selection thereby increasing the biological fitness associated with having specific genetic mutations.

Once the behavioral practice of regular adult consumption of animal milk was in place in these diverse groups, concomitant changes in the gene expression patterns of the domesticated livestock were required for the enhanced production of the additional milk needed to meet the dietary demands of adult human consumers. We now know that there is substantial geographic coincidence between high diversity in cattle milk genes, locations of the European Neolithic cattle farming sites (>5,000 years ago) and present-day LP among adult Europeans (Beja-Pereira et al., 2003). The reciprocal influences of genetics and the cultural environments in these early pastoralists facilitated the subsequent social and behavioral shifts observed as a result of this changed dietary pattern. In certain populations of domesticated cattle and humans their genes had coevolved in response to changes in behavior that became imbedded in the emerging social fabric of these selected groups.

Genetics has now been able to provide researchers with the specific molecular variants involved in each of the geographical regions that this coevolutionary pattern has emerged and their calculated emergence times [discussed in Ingram et al. (2009)]. These data have, in turn, allowed us to refine and expand our interpretations of the social consequences of these behavioral shifts and their ramifications on regional development and health, reemphasizing the dynamic feedback loop between “genes” and “environment.”

In European dairying groups and their descendants, a single genetic mutation explains the distribution of the LP phenotype [see Anagnostou et al. (2009)]. This single nucleotide polymorphism is located 13.9 kb upstream from *LCT* (13910T) was proposed to be the cause for LP. The -13910^∗^T allele, which is widespread in Europe was found to be located on an extended haplotype of 500 kb or more (Anagnostou et al., 2009). In Central Asia the causal polymorphism for LP is the same as in Europe (-13.910C > T, rs4988235; Heyer et al., 2011), suggesting genetic diffusion between the two geographical regions.

However, elsewhere among milk-consuming groups, several other mutations are associated with LP. For example, in some South Africans, the -14010^∗^C allele, is associated with LP (Jensen et al., 2011). In East Africa, the primary mutation appears to be (-13907G; Enattah et al., 2008). The European (-13910T) and the East African (-13907G) allele share the same ancestral background and most likely a similar social and behavioral history, probably related to the same cattle domestication event. In Saudi Arabia, the European allele (-13910T) is absent and instead two new mutations are found as a compound allele: (-13915T/G) within the -13910 enhancer region and a synonymous SNP in the exon 17 of *MCM6* (-3712T/C), -3712 base pairs from *LCT* (Enattah et al., 2008). This compound allele is highly prevalent among many Arab populations and shows a different, highly divergent ancestral haplotype, perhaps in response to camel domestication and milk consumption (Enattah et al., 2008).

Recent studies in Tibet (Peng et al., 2012), however, suggest that three novel SNPs (-13838G/A, -13906T/A, and -13908C/T) are responsible for LP among Tibetans, placing them at variance with European, East African, and Middle Eastern genetic patterns. This emphasizes the concept of local geographic-based responses to common environmental challenges and reinforces the need for taking population history into consideration in the search for genes responsible for health-related conditions.

The worldwide geographical variability in the genetics underlying the LP phenotype suggests that this phenotype emerged independently several times in human history. Furthermore, several independent mutations tied to regional social events coupled with local behavioral choices and existing patterns of genetic variation are involved in its current multiethnic distribution. These genetic data can both broaden and deepen the scope of environmental analyses of LP by permitting researchers to identify the origins of this phenotype in any particular individual or group and to correlate a specific genetic variant of LP with the unique historical and evolutionary circumstances that characterize a specific milk-consuming group.

## CASE STUDY: AUTISM SPECTRUM DISORDERS (ASDs): ENVIRONMENTAL COMPLEXITY AND DYNAMISM IN GENE EXPRESSION

One of the most poignant examples of environment interactions influencing important clinical abnormalities are the autism spectrum disorders (ASDs). ASDs are a complex group of neurodevelopment disorders, which are still poorly understood (Centers for Disease Control and Prevention, 2012). ASDs typically appear before 3 years of age, and affect the brain’s normal development of social and communication skills. ASDs appear to be rising in frequency and are refractory to current treatments. In 2008, the overall estimated prevalence of ASDs was 11.3 per 1,000 (one in 88) children aged 8 years. Overall ASD prevalence estimates varied widely geographically and ASD prevalence estimates also varied widely by sex and by biosocial/ethnic group (Centers for Disease Control and Prevention, 2012).

From studies of both monozygotic and dizygotic twins, it is evident that identical twins are much more likely than fraternal twins or siblings to both have autism. Similarly, language abnormalities are more common in relatives of autistic children. Chromosomal abnormalities and other neurological problems are also more common in families with autism. Genetic factors clearly play a role as well in ASDs. Some of the recently proposed genetic factors in ASDs include *DRD2* and *PPP1R1B* in males (Hettinger et al., 2012), chromosomal (Yang et al., 2012), copy number variations in candidate genes (Griswold et al., 2012), microRNAs (miRNAs) associated with brain development and maturation (Mellios and Sur, 2012), gene-disrupting mutations (nonsense, splice site, and frame shifts; Iossifov et al., 2012), deficient GABA neurotransmission (Mendez et al., 2012), and functional mutations in postsynaptic scaffolding proteins at excitatory synapses (Ting et al., 2012).

Extensive research has been so far unable to explain the etiology of ASDs, whereas a growing body of evidence suggests that the involvement of local environmental exposures to specific, potent bioactive factors plays a major role. Phthalates, given their extensive use and their persistence, are ubiquitous environmental contaminants implicated in the etiology of ASDs. These are endocrine-disrupting chemicals suspected to interfere with neurodevelopment. Therefore, they represent interesting candidate environmental risk factors for ASDs pathogenesis (Testa et al., 2012). Some other recently suggested environmental triggers for ASDs include prenatal ethanol exposure (Middleton et al., 2012), mercury poisoning, changes in the digestive tract, diet (Pennesi and Klein, 2012), oxidative stress (Frustaci et al., 2012), maternal fever during pregnancy (Zerbo et al., 2012), paternal age (Eriksson et al., 2012; Iossifov et al., 2012), inadequate breast feeding (Al-Farsi et al., 2012), and persistent exposure to organic polybrominated diphenyl ethers (PBDEs) used in commercial flame retardants (Woods et al., 2012).

Overall, the behavioral polytypism associated with ASDs suggests that an array of genetic and environmental factors influence the individual autism phenotype [see Buchholz et al. (2013)]. The problem for scientists working on ASDs is that this spectrum of disorders are rarely diagnosed in children younger than 2 years, since diagnosis is based entirely on behavioral tests (Frustaci et al., 2012). Changes in the epigenome (Rangasamy et al., 2013) can serve as indicators of these gene–environmental interactions and help clarify the contributions of each set of factors (individually and interactively) to autism [see Hall and Kelley (2013)].

As mentioned, environmental factors that mediate epigenetic changes appear to play a strong role in the etiology of ASDs (Ladd-Acosta et al., 2013). Unbiased investigations of a multitude of novel candidate genes encoding nuclear factors implicated in chromatin remodeling, histone demethylation, histone variants, and chromatin alterations suggest that DNA methylation underlies many neurodevelopmental aberrations, including the ASDs (LaSalle, 2013a). Additionally, long non-coding RNA (lncRNAs) contribute to ASDs risk (Wilkinson and Campbell, 2013). Future analyses of these non-coding RNAs as well as studies of a variety of epigenetic modifications around genetic risk factors augmented with quantified measures of environmental exposures and methylome analyses are expected to be important for understanding the complex etiology of autism (Hu, 2013; LaSalle, 2013b).

## CASE STUDY: METABOLIC SYNDROME (MetS): REDEFINING THE PARAMETERS OF POPULATION BEHAVIORAL AND BIOLOGICAL SUBSTRUCTURE:

The most common diseases affecting the US population are complex disorders that develop as a result of defects in multiple genetically controlled systems in response to environmental challenges (Berdasco and Esteller, 2013). MetS represents a set of disorders that frequently occur together and increase the risk of further cardiovascular and metabolic disease. MetS is a global pandemic of enormous medical, economic, and social concern affecting a significant portion of the world’s population (Alberti et al., 2009). A number of organizations have developed definitions for the MetS to guide clinician and researchers in identifying the syndrome. One of the earliest was the World Health Organization in 1998 (Grundy, 2007). Later, the National Cholesterol Education Program/Adult Treatment Panel III (NCEP/ATPIII) developed diagnostic criteria for the MetS (Farook et al., 2012; Forti et al., 2012). The International Diabetes Federation and Joint Interim Statement also proposed criteria for identifying MetS (Ruemmele and Garnier-Lengliné, 2012).

All of these definitions of MetS include abdominal obesity, hyperglycemia, hypertriglyceridemia, low high-density lipoprotein cholesterol, and hypertension (Ruemmele and Garnier-Lengliné, 2012) and are considered the five indicators of the MetS. If an individual possesses three or more of the five risk indicators, then that person is defined as having MetS and is said to be at increased risk for the development of cardiovascular disease and also for Type 2 diabetes mellitus. Details of these five risk indicators include: elevated triglyceride levels (≥150 mg/dl), elevated systolic (≥130 mmHg) and diastolic (≥85 mmHg) blood pressures, elevated fasting blood glucose level (>110 mg/dl), reduced blood levels of High Density Lipoproteins (<40 mg/dl for men) and <50 mg/dl for women), and abdominal obesity [large waist circumference (WC), initially defined as >102 cm for men and >88 cm for women].

However, these definitions were developed from studies of Europeans (and their descendants; Wang et al., 2009) and the cut-off values were promulgated as universal until recently when studies of Chinese and Koreans demonstrated that some East Asians develop diabetes and several risk indicators of cardiovascular disease at lower body mass index (BMI) and WC values than do European-descended populations (Zorzi et al., 2009; Kim et al., 2012). This has given rise to several new studies that result in our now suggesting that BMI and WC cut-offs be made more population-specific (Grundy, 2007). Although the cut-off values are still subject to change with additional research, most Asians (whether East or South Asians) have lower BMI and WC cut-off values than do European Americans or other Europeans when they begin to express the abnormal clinical indicators of MetS. Alternative patterns for MetS may also exist for aboriginal Canadian AmerIndians (Nikitin et al., 2012). This additional biological information on human diversity in BMI and WC may call into question the reports, in Siberia and other East Asian regions, for example, that the overall prevalence of both diabetes and MetS among the indigenous East Asian population is lower than among Europeans (Roth and Sathyanarayana, 2012). It may simply be that the detection criteria are different and that some East Asian cases are being missed using European-based physiological definitions.

Different BMI and WC cut-offs suggest that human populations are highly variable and that diet and other environmental factors have a huge impact on body shape, body composition, and susceptibility to disease. This is not unexpected since signaling peptides produced in peripheral tissues such as gut, adipose tissue, and pancreas communicate with brain centers, such as hypothalamus and hindbrain to manage energy homeostasis and these processes are highly sensitive to the influence of environmental variables (Du et al., 2012). The regulatory mechanisms of energy intake and storage have evolved during extended periods of famine in human evolution to allow for optimal adaptation and ultimately protect the species from extinction. It is now clear that these circuitries are influenced by prenatal and postnatal environmental factors including dietary adequacy and endocrine disruptive chemicals (Du et al., 2012).

Hypothalamic appetite regulatory systems develop and mature in utero and early infancy, and involve signaling pathways that are important also for the regulation of the onset of puberty (Du et al., 2012). Metabolic pathways involved in the regulation of growth, body weight gain, and sexual maturation are largely affected by epigenetic programming that can impact both current and future generations (Du et al., 2012). Therefore, researchers studying MetS cross-culturally would be well served to integrate both ecological and evolutionary perspectives in their investigations since population substructure can dramatically influence the initiation of MetS’s downstream events. In particular, intrauterine and early infantile developmental phases are highly plastic and susceptible to factors that affect metabolic programming. These, therefore, affect metabolic function throughout the lifespan [discussed in Du et al. (2012)]. Thus, the underlying mechanisms regulating human metabolic biology are likely to be evident in many different groups although the specific genes involved will vary between geographical groups. Definitions of metabolic disease indicators should not be generalized to all world populations, particularly when the standards are disproportionately based a minority of the world human biodiversity and are largely restricted to measurements on individuals from economically developed regions.

Although genetics, physiology, and environmental components play a major role in the onset of the MetS diseases due to excessive fat accumulation, little is known about how or to what extent each of these factors contributes to the actual syndrome (Alberti et al., 2009) and which specific genes may serve as triggers. Currently, the search for patterns in the genetics of MetS is in its infancy. What has emerged is that a host of different genes on different chromosomes in different human groups that appear to be associated to varying degrees with MetS. For example, in Mexican Americans, chromosome 7q21 shows strong evidence for linkage to MetS, especially between markers D7S2212 and D7S821 (Forti et al., 2012). Six chromosomal regions exhibit potential evidence for linkage with MetS in this group. 29 single-nucleotide polymorphisms (SNPs) from the fatty acid translocase [*FAT* or *CD36*, 18 SNPs] and guanine nucleotide binding protein and α transducing 3 [*GNAT3*, 11 SNPs] gene (Forti et al., 2012), are associated with MetS and its related traits in Mexican Americans. In this same population, several SNPs were also associated with MetS and its related traits with the strongest associations being linked with rs11760281 in *GNAT3* and rs1194197 near *CD36* (Forti et al., 2012). This accounted for approximately 18% of the MetS linkage on chromosome 7q21 and together these genetic changes conferred nearly a threefold increase in MetS risk for Mexican Americans (Forti et al., 2012).

Among Han Chinese (Dujic et al., 2012), however, an association study suggests that *ADIPOQ* variants are associated with the risk of MetS and include genotypes rs266729CG; rs1063539GC, GC/CC; rs16861205AA, and rs7649121AT. Functional studies of these molecular variants of *ADIPOQ* are still pending.

In a recent study of Bosnian (Eastern Europe) children with MetS, a common rs45487298 polymorphism in *HSD1181* is thought to possibly have a protective effect against insulin resistance (Min et al., 2012). In an effort to identify the gene networks involved in MetS in British Europeans, researchers conducted Min et al. (2012) and Alkharfy et al. (2012) conducted whole-genome expression and genotype profiling and found nine coexpresssed genes in abdominal tissue and six in gluteal adipose tissue, but no coexpressed genes in whole blood. The rs10282458 polymorphism, affecting expression of *RARRES2* (encoding chemerin), was associated with BMI and rs2395185, affecting inter-depot differences of HLA-DRB1 expression, was associated with high-density lipoprotein and the BMI-adjusted waist-to-hip ratio in this British population.

Among Saudi Arabians, a significant association of 894G > T, 4a/b, and -786T > C polymorphisms with MetS in *eNOS* is evident (Csaba, 2011). A genetic predisposition to develop abnormal metabolic phenotypes, consistent with an increased prevalence of metabolic phenotypes has reportedly been detected in the Saudi Arab population with these genetic polymorphisms. Researchers have also reported a high prevalence of MetS among Kuwaiti adults (Al Zenki et al., 2012), presumably with a similar genetic or environmental basis.

From this cursory overview of gene-association studies, it appears that great diversity exists in specific susceptibility genes that characterize the genetic underpinnings of MetS across geographical groups (see da Costa et al., 2014). Epigenetically, MetS seems to reflect some level of hormonal imprinting (Lacaria et al., 2012) which, when faulty can produce disturbances in methylation patterns. CNVs also appear to be involved in some cases of MetS (Wang et al., 2009; Rottiers and Näär, 2012). Dysregulation of miRNA may also contribute to the metabolic abnormalities associated with MetS and thus may provide a potential source of cardiometabolic therapeutic targets.

As the technology continues to improve, the ability to integrate these epigenetic data into the analyses of MetS should prove extremely important, permitting more precise assessments of the epigenetic consequences of particular events on MetS phenotypes [see Turcot et al. (2012) and Wang et al. (2012)].

## STRATEGIES FOR DEVELOPING NEW PARADIGMS AND RESEARCH METHODOLOGIES

Epigenetic factors that control chromatin dynamism are essential for the proper functioning of tissue homeostasis, cell identity, and development. Deregulation of epigenetic profiles is intimately associated with metabolic pathologies and neurological diseases, among other complex disorders (Cox et al., 2013). From the case studies presented here, we can see that both our assumptions about the “gene” and the “environment” as well as their interactions need to be more nuanced, more localized to the specific populations under consideration, and more sensitive to the particular relevant local environmental factors of influence.

## Conflict of Interest Statement

The author declares that the research was conducted in the absence of any commercial or financial relationships that could be construed as a potential conflict of interest.
